# Accurate modelling of intrabeam scattering and its impact on photoinjectors for free-electron lasers

**DOI:** 10.1038/s41598-026-36558-3

**Published:** 2026-01-21

**Authors:** Thomas G. Lucas, Paolo Craievich, Eduard Prat, Sven Reiche, Erion Gjonaj

**Affiliations:** 1 Paul Scherrer Institut, Forschungsstrasse 111, 5232 Villigen, Switzerland; 2 Institute for Accelerator Science and Electromagnetic Fields (TEMF), TU Darmstadt, Darmstadt, Germany

**Keywords:** Intrabeam scattering, Injector, Electron source, Free-electron laser, Engineering, Optics and photonics, Physics

## Abstract

Intrabeam scattering (IBS) is a fundamental effect that can limit the performance of high-brightness electron machines but has so far been neglected in standard modeling of RF photoinjectors. Recent measurements at SwissFEL show that the slice energy spread (SES) in the injector is significantly underestimated in standard beam dynamic simulations. In this paper, we employ a dedicated Monte Carlo simulation model that accurately predicts IBS-induced SES growth in the photoinjector of an X-ray free-electron laser. The simulations are benchmarked against SES measurements at the SwissFEL and are supported by a new analytical model. The results show that IBS-induced SES growth occurs throughout the injector, most prominently in the electron source, and must be included in performance assessments. We further demonstrate that while 5D brightness is largely conserved, the 6D brightness degrades with propagation, highlighting the need to account for IBS in accurate photoinjector design and optimization.

## Introduction

High-brightness electron beams are critical to the lasing performance of X-ray Free-Electron Lasers (XFELs). The brightness of the beam is defined as the product of the bunch charge and inverse phase-space volume. According to Liouville’s theorem, the 6D brightness at the cathode sets the theoretical upper limit downstream the accelerator, making its preservation throughout the injector and linac a primary objective in the design and operation of XFELs.

At SwissFEL, measurements of the slice energy spread (SES) in the injector^1,2^ revealed values significantly higher than those predicted by standard tracking codes such as ASTRA^3^, GPT^4^, and OPAL^5^. Similar discrepancies have been observed at other facilities, including the European XFEL and PITZ^6,7^. These discrepancies have been attributed to physical effects not captured by standard macroparticle-based space-charge models, most notably the microbunching instability (MBI) and intrabeam scattering (IBS). A subsequent measurement campaign at SwissFEL to disentangle their respective contributions found that IBS contributed approximately 6 keV to the total of 15 keV SES measured in ^2^, with the remaining contribution primarily driven by MBI. However, an accurate means to model IBS was not available during these measurements, rather a scaled model from Piwinski with amendments from Huang was employed^2,18^.

The SES of the electron beam is critical for XFEL operation, as the relative SES must remain below the Pierce parameter for effective lasing ($\rho \sim 10^{-4} - 10^{-3}$ for X-rays) ^8^. Furthermore, a higher SES of the beam limits the minimum pulse length, restricts the achievable wavelengths, and demands greater seed power in seeded FEL schemes. Consequently, accurate SES modelling along the injector and linac is essential to accurately determine machine performance^2^.

In this paper, we present a Monte Carlo–based IBS model implemented in the particle-tracking code REPTIL^9^ together with a complementary analytical IBS model, and validate each against the measurements at SwissFEL. These tools are used to study SES and 6D brightness evolution along the injector, emphasising the electron source. Our results highlight the importance of including IBS in the design of next-generation high-brightness XFEL electron sources and injectors. It will be shown that the concept of developing an electron source strictly around the concept of high 5D brightness may not necessarily lead to an overall increase in performance, contrary to commonly used methods.

## Role of 6D brightness in XFEL performance

The performance of an XFEL is characterised by the FEL (or Pierce) parameter, $\rho$, which in 1D-FEL theory is given by^12^

1 $$\begin{aligned} \rho := \frac{1}{\gamma }\bigg [\bigg (\frac{K f_c}{4 k_u \sigma _x} \bigg )^2 \frac{I_p}{2 I_A} \bigg ]^{1/3}, \end{aligned}$$

where $\gamma$ is the Lorentz factor, *K* the undulator parameter, $k_u$ the undulator wavenumber, $f_c\approx 1$ the coupling factor, $I_p$ the peak current, $I_A$ the Alfvén current, and $\sigma _x$ the rms beam size. Assuming cylindrical symmetry and defining $B_{5D}:= \frac{2I_p}{\epsilon _N^2}$, with $\epsilon _N$ the normalised transverse emittance, we find^12^

2 $$\begin{aligned} \rho \propto B_{5D}^{1/3}. \end{aligned}$$

While this 1D theory highlights key dependencies, it neglects critical effects such as beam diffraction and energy spread. In particular, Landau damping reduces FEL gain when $\frac{\sigma _\gamma }{\gamma }> \rho$, with $\sigma _\gamma$ the SES. In the limiting case $\frac{\sigma _\gamma }{\gamma } \approx \rho$, Eq. (1) becomes

3 $$\begin{aligned} \rho ^2 \frac{\sigma _\gamma }{\gamma } \approx \frac{1}{I_A \gamma ^3} \bigg (\frac{K f_c}{4 \sqrt{2} k_u} \bigg )^2 \frac{I_p}{\sigma _x^2}. \end{aligned}$$

Using $\sigma _x = \sqrt{\beta \epsilon _N/\gamma }$ where $\beta$ is the optical beta function, this can be rewritten as

4 $$\begin{aligned} \rho ^2 \frac{\sigma _\gamma }{\gamma } \approx \frac{1}{I_A} \bigg (\frac{K f_c}{4 \sqrt{2} \gamma k_u} \bigg )^2 \frac{I_p}{\epsilon _N \beta }. \end{aligned}$$

Furthermore, the FEL performance is affected by emittance-induced phase oscillations, as well as by the SES of the beam. In this limiting case, within a strong focusing undulator, the FEL parameter can be approximated by^13^

5 $$\begin{aligned} \rho \approx \frac{\epsilon _N \lambda _u}{4 \lambda \gamma \beta }, \end{aligned}$$

where $\lambda = \frac{\lambda _u}{2 \gamma ^2} (1+K^2/2)$ is the resonant wavelength and $\lambda _u$ is the undulator wavelength. Substituting Eq. (5) into Eq. (4), and defining the 6D brightness as $B_{6D}:= \frac{2I_p}{\epsilon _N^2 \sigma _\gamma }$, we find

6 $$\begin{aligned} \rho \propto B_{6D}. \end{aligned}$$

Thus, the FEL gain is ultimately proportional to the full 6D-brightness. This highlights the importance of accurately modelling the longitudinal beam dynamics, including effects such as IBS, which give rise to SES-growth along the accelerator line.

## Modelling of IBS in the SwissFEL injector

 Intrabeam scattering occurs on timescales that are significantly shorter than the time steps typically used in such particle tracking simulations and also arises from interactions between individual particles. As a result, its effect is largely missed by standard macroparticle-based simulation approaches. In this section, we present two complementary approaches to modelling intrabeam scattering, namely an analytical formalism and a numerical model, which enable more accurate predictions of the SES evolution in the SwissFEL injector.

**Fig. 1:**
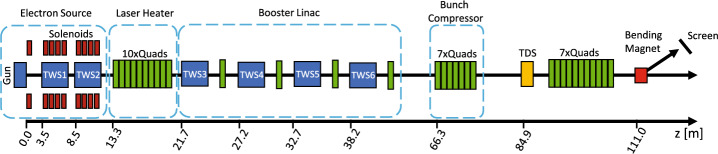
Layout of the SwissFEL injector from the SES measurements^2,14,^ which is used in tracking simulations. In the measurement setup considered in this work, the R$_{56}$ of the laser heater and bunch compressor are set to zero.

Our particle tracking simulations are performed using the REPTIL code^9^, and model the full SwissFEL injector from the photocathode up to the diagnostics beamline at 111 m (Fig. 1). All RF and magnetic elements are taken into account while the laser heater and bunch compressor have an $R_{56}=0$ to properly reflect the conditions used during the measurements to mitigate the MBI contribution to the SES^2^. For the calculations described in the following sections, the analytical calculations are employed upon standard macroparticle-based, space-charge–only simulation data, whereas the numerical simulations use an additional IBS module implemented in the REPTIL tracking code, that will be described below.

For these simulations, the momentum distribution of the bunch emitted from the cathode is modelled as a simple isotropic emission across an area optimised for best machine performance. This emission model leads to the intrinsic transverse emittance on the cathode of 0.55 mm mrad/mm, which has been validated by measurements^17^. This initial momentum distribution drives an initial SES whose range is approximately 0.230 eV. We’ll see below that this is negligible compared to SES induced along the electron source.

### Analytical model

For our analytical approach, we introduce a new model based on Piwinski’s IBS-model that is applicable in the case of a low-energy injectors, following on from^18^. In this reformulation, Huang modified Piwinski’s model for linacs by neglecting synchrotron oscillations. The SES growth for a 3D Gaussian bunch over a distance $\Delta s$ is given by:

7 $$\begin{aligned} \sigma _\gamma ^2 = \sigma _{\gamma ,0}^2 + \frac{r_e^2 N_b \Lambda _c}{4 \sigma _x \epsilon _N \sigma _z} \Delta s, \end{aligned}$$

where $\sigma _{\gamma ,0}$ is the initial SES, $r_e$ the classical electron radius, $N_b$ the number of electrons, $\sigma _z$ the rms bunch length, and $\Lambda _c$ the Coulomb logarithm. To account for the local nature of IBS, we will use the mean slice size over x and y ($\sigma _{r,s}=\sqrt{\sigma _{x,s}\sigma _{y,s}}$) and the slice normalised emittance ($\epsilon _{N,s}$) of the *slice* instead of the corresponding rms-valued quantities used in^18^. Since IBS in our case is dominated by particle collisions in transverse planes (whereas the longitudinal space-charge interaction is negligible), this slice-based treatment is more appropriate. In particular, our measurements mention the SES of the *central slice* of the SwissFEL bunch, therefore we use the parameters of this particular slice. We can also relate $N_b/\sigma _z$ to the peak current as

8 $$\begin{aligned} N_b/\sigma _z = \sqrt{2 \pi } \frac{q_s}{c e \Delta z_s} = \frac{\sqrt{2 \pi } I_p}{c e} \end{aligned}$$

where $q_s$ is the charge in the central slice, $\Delta z_s$ is the full slice length and $I_p$ is the peak current. In the above, we have modelled the central slice as a Gaussian beamlet of rms length $\sigma _{z,s}=\Delta z_s/\sqrt{2\pi }$. This allows the direct application of Piwinski’s theory for Gaussian distributions in Eq. (7) to the IBS growth rate within a single slice of the bunch. Notably, the $\sqrt{2 \pi }$ factor naturally recovers the empirical correction factor of 2.4 used in^2^. Substituting Eq. (8) into Eq. (7) yields:

9 $$\begin{aligned} \sigma _\gamma ^2 = \sigma _{\gamma ,0}^2 + \frac{\sqrt{2 \pi } r_e^2 I_p \Lambda _c}{4 c e \sigma _{r,s} \epsilon _{N,s}} \Delta s. \end{aligned}$$

The Coulomb logarithm is evaluated locally for the slice following the argument in^18^ as:

10 $$\begin{aligned} \Lambda _c = \ln \left( \frac{\Delta \gamma _{\max }}{\Delta \gamma _{\min }}\right) , \quad \Delta \gamma _{\max } = \gamma ^2 \sigma _{r',s}, \quad \Delta \gamma _{\min } = \frac{r_e}{\sigma _{r,s} \sigma _{r',s}}. \end{aligned}$$

where $\sigma _{r',s}$ is the mean slice beam divergence. Using Eq. 9, we may calculate the total SES now with contributions by IBS using beam parameters obtained from standard space-charge, macro-particle tracking simulations.

### Numerical simulation model

Recently, IBS models have been implemented in the tracking codes REPTIL^9^ and RF-Track^10,11^. The former has been used to model a simplified version of the European XFEL injector. In this section, we outline our approach to incorporating IBS in beam dynamics simulations of the SwissFEL injector using the REPTIL code.

To simulate collisional effects, we employ a stochastic Monte Carlo model in the REPTIL tracking code, used here specifically for IBS modelling in XFEL injectors. The technique has been thoroughly described in^9^. The model is based on Nanbu’s cumulative binary collision method^15^, where particles are grouped into spatial cells, and random pairs within each cell undergo effective Coulomb collisions. These collisions are ultimately modelled as momenta rotations in the centre-of-mass frame of the particle pair.

The scattering angle distribution of the binary collision process is determined by the local Coulomb logarithm, which REPTIL computes dynamically using the evolving charge density and momenta distributions^9^. This enables accurate IBS modelling for arbitrary distributions. This is particularly important in photoinjector simulations, where the beam distribution along the beamline can vary significantly.

**Fig. 2:**
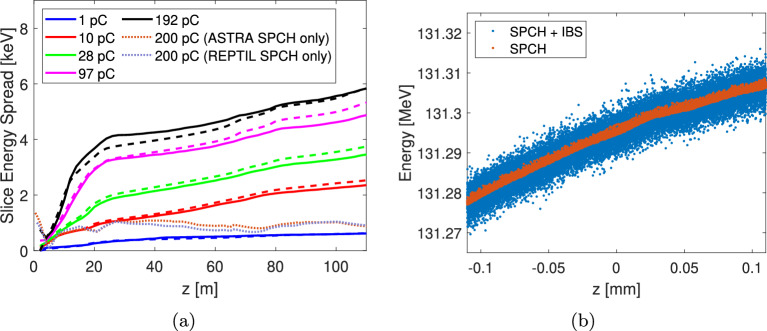
(**a**) Slice energy spread over the SwissFEL injector, modelled in REPTIL with the IBS module (solid lines) and using the analytical model (dashed line) for the five different bunch charges used in the measurements^2^. For comparison, the SES calculated for the SwissFEL baseline case (200 pC) using standard space-charge–only macroparticle tracking simulations in ASTRA and REPTIL, without IBS, is also shown (dotted lines). (**b**) A zoom of the longitudinal phase space of the bunch at $z = 13$ m with (blue) and without (red) IBS included in the space-charge (SPCH) simulations performed using the REPTIL code.

### A comparison between modelling and measurements

For accurate comparisons to the measurements, our modelling of the SES along the injector used the same bunch charges as in the measurements presented in^2^. To the calculate slice values, the slices are defined by the longitudinal distribution, of which we take the spatially central slice as done in the measurements. The number of slices was chosen such that the total energy spread of the central slice converged with an increasing number of slices. To match the measurements, the laser spot size and duration on the cathode within the simulations remained fixed, with only the laser intensity varied to control the bunch charge. The distribution of the laser was uniform in the transverse plane and Gaussian in the longitudinal plane.

**Fig. 3:**
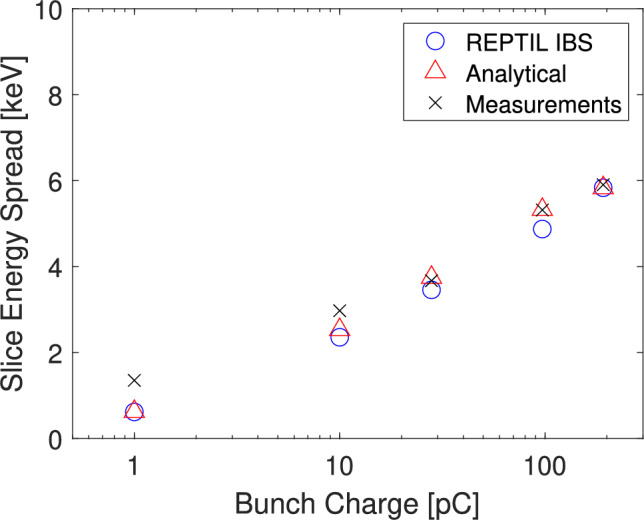
Comparison between the measurements at the SwissFEL, the numerical simulations with IBS performed with REPTIL and the analytical calculation using Eq. (9). The values are given at 111 m downstream of the cathode.

Figure 2a depicts the calculated SES using both the analytical model (dashed line) and numerical model (solid line) for each of the charge cases where a strong agreement between the numerical and analytical model is found.

The most notable feature of the modelled SES is its fast growth in the electron source region ($z<13$ m), which slows down only when the beam enters the booster linac. This is explained by the growth of the transverse beam size, $\sigma _{x,y}$, which occurs at the end of the electron source.

Furthermore, Fig. 2a also presents the SES calculated with standard space-charge simulations using ASTRA and REPTIL at the nominal SwissFEL baseline charge of 200 pC, where we also see good agreement between the two codes. Comparing the same charge situations at 111 m with and without IBS shows that the IBS-induces SES growth is approximately an order of magnitude larger when including IBS than that predicted by standard space-charge macroparticle-based tracking codes^1^.

This systematic underestimation of the SES in standard space-charge codes results in a predicted 6D brightness that is nearly an order of magnitude higher than that obtained in measurements. Consequently, accurate modelling of IBS is essential for a realistic assessment of the XFEL performance.

Figure 2b illustrates the impact of IBS by comparing longitudinal phase space at $z=13$ m for the REPTIL space-charge (SPCH) simulations with and without the IBS module. IBS introduces notable energy diffusion, broadening the local energy spread while preserving the overall energy curvature.

To validate the analytical and numerical models, Fig. 3 compares the modelled values to the measurements performed at SwissFEL across these various bunch charges. We observe a similarly strong agreement between the SES measurements, and the modelled values validating the two methodologies.

## Impact of IBS on the brightness of electron sources

As shown in Eq. (9), increasing peak current or reducing slice emittance (which are the hallmarks of high 5D brightness beams) also amplifies IBS-induced SES. This raises concerns about evaluating new electron sources on the basis of their 5D brightness, which is often used in the literature as the figure-of-merit for the beam quality. In^19^, a new high-brightness electron source was proposed to generate a beam of higher 5D brightness from the cathode. Here, we assess the impact of IBS on this design in comparison with the existing SwissFEL RF photogun. While our study focuses on these two electron sources, IBS effects are expected to be relevant for all high-brightness electron sources and photoinjectors.

**Fig. 4:**
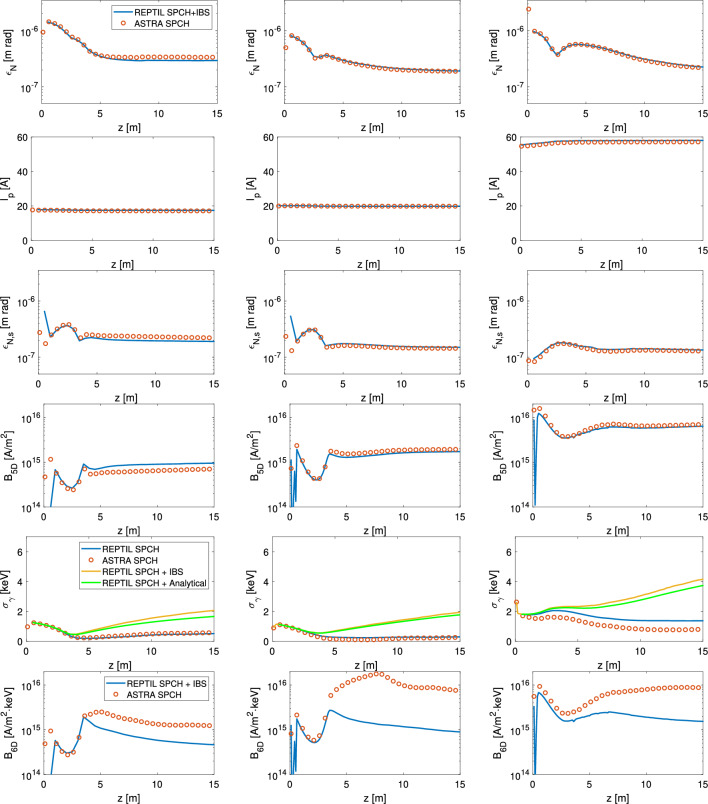
Evolution of the projected emittance, peak current, central slice emittance, 5D brightness, SES and 6D brightness (top to bottom) calculated for the SwissFEL RF photogun with Gaussian longitudinal distribution (left), SwissFEL RF photogun with uniform longitudinal distribution (middle) and a proposed higher brightness travelling-wave photogun with uniform distribution (right). The project emittance, peak current and slice emittance are simulated with REPTIL and bench-marked against ASTRA. The SES was calculated with a standard space-charge only simulation (SPCH), using the IBS module of REPTIL (SPCH + IBS) and the analytical model in Eq. (9) (SPCH only with analytical IBS).

We perform numerical tracking simulations using the REPTIL code and benchmark these against ASTRA, which is a widely validated tool. Three scenarios are considered: (a) the SwissFEL gun with Gaussian longitudinal distribution for the photocathode laser, (b) the SwissFEL gun with a uniform longitudinal distribution for the photocathode laser, and (c) the proposed traveling-wave (TW) gun with a uniform longitudinal distribution for the photocathode laser. This allows us to examine the role of the initial longitudinal distribution of the electron bunch (Gaussian vs. uniform) on IBS. Furthermore, we can assess how the beam brightness is affected by IBS and, furthermore, also validate the analytical model for different initial distributions. The latter is important as it is common to optimise an electron source with the idealised uniform distribution although such a longitudinal distribution is difficult to achieve practically.

For these three cases, REPTIL simulations are run with the standard space-charge (SPCH) model and with the space-charge model with IBS enabled (SPCH+IBS). All simulations include the electron source up to 15 m, as shown in Fig. 1, to capture beam evolution along *z* for the electron source component of the injector.

Figure 4 compares the central slice parameters, as well as the projected emittance, for the three cases. The slice emittance and peak current remain nearly constant along *z*, indicating that these beam qualities are established early in the electron source and preserved by rapid acceleration. This is consistent with the nearly constant 5D brightness derived from these parameters, observed downstream of the point where slice-mixing subsides (approximately 5 m downstream of the cathode). The effect of IBS on the emittance and peak current was found to be negligible compared to the effects of space-charge and within the resolution of the plots, so only the SPCH+IBS curve of REPTIL is shown.

Figure 4 also shows the SES evolution for both electron sources using standard SPCH simulations (blue), SPCH+IBS simulations (yellow), the analytical model of Eq. (9) applied to slice parameters from the SPCH runs (green) and the ASTRA simulation as a reference (red). We observe a strong growth in the SES at the start of the first accelerating structure (approximately 3 m) when including IBS, which is not found using standard space-charge modelling. Furthermore, we can use the SES to understand the machine performance that above was illustrated to scale with the 6D brightness. Unlike the 5D brightness, the 6D brightness decreases along *z* due to IBS-driven SES growth leading to a deterioration of beam quality along *z*. This is observed in all electron source cases. When comparing the S-band SW and C-band TW electron guns for 6D brightness, we see that the TW gun continues to have a greater brightness than the existing SwissFEL gun. However, performance improvement for this new electron source is less than originally anticipated based on the estimation of the 5D brightness^19^. This effect is due to the presence of IBS, which so far had been omitted in the analysis.

Finally, we investigate the role of the initial bunch distribution on IBS-induced SES. Monte Carlo simulations for both Gaussian and uniform distributions agree well with the analytical model, showing that the choice of initial laser distribution has little impact on the analytical calculation compared to slice parameters such as peak current, bunch size, and emittance. However, for precise evaluation of distribution-dependent effects, Monte Carlo–based IBS calculations remain preferable.

In electron sources, IBS must be included for accurate modelling of the six-dimensional phase space. Owing to the stringent requirements for emittance compensation, IBS is expected to be difficult to mitigate and therefore represents a fundamental limitation in electron sources.

## Conclusions

Recent SES measurements at SwissFEL confirm that IBS plays a key role in limiting 6D beam brightness. This effect is typically not included in standard beam dynamics simulations. For the analysis in this paper, we apply first-principle beam dynamics simulations including particle collision effects. In addition, we develop an accurate analytical IBS model, based on Piwinski’s theory, which is better suited for electron injector calculations. In both cases, we obtain excellent agreement with the measurement data for the SwissFEL injector reported in^2^.

IBS has the greatest impact in the early acceleration phase, especially in the electron source. Simulations of both the current SwissFEL electron source and a prospective upgrade show that while 5D brightness is largely conserved along *z* at the injector, 6D brightness decreases due to IBS-driven SES growth. This underscores the need to account for IBS effects in electron source design, particularly in the case that performance is strongly SES-dependent, such as seeded-FELs or where strong bunch compression is required.

With our improved understanding of IBS, the next step is to fully model the SwissFEL injector up to the bunch compressor, incorporating our proposed electron source upgrades and accounting for IBS effects. At the bunch compressor we expect the SES to increase linearly with the compression factor therefore accurate modelling of the IBS is key.

## Data Availability

All data supporting the findings of this study are available upon reasonable request to Thomas G. Lucas (thomas.lucas@psi.ch).
